# Could Temperamental Features Modulate Participation in Clinical Trials?

**DOI:** 10.3390/jcm12031121

**Published:** 2023-01-31

**Authors:** Simona Cintoli, Camilla Elefante, Claudia Radicchi, Giulio Emilio Brancati, Silvia Bacciardi, Joyce Bonaccorsi, Gabriele Siciliano, Icro Maremmani, Giulio Perugi, Gloria Tognoni

**Affiliations:** 1 Neurology Unit, Santa Chiara University Hospital, 56126 Pisa, Italy; 2 Psychiatry Unit, Department of Clinical and Experimental Medicine, University of Pisa, 56126 Pisa, Italy; 3 Institute of Neuroscience, National Research Council, 56124 Pisa, Italy; 4 Department of Psychiatry, North-Western Tuscany Region NHS Local Health Unit, Versilia Zone, 55049 Viareggio, Italy; 5 PISA-School of Clinical and Experimental Psychiatry, 56100 Pisa, Italy; 6 Neurology Unit, Department of Clinical and Experimental Medicine, University of Pisa, 56126 Pisa, Italy; 7 G. De Lisio Institute of Behavioral Sciences, 56127 Pisa, Italy; 8 Saint Camillus International University of Health and Medical Sciences (UniCamillus), 00131 Rome, Italy

**Keywords:** Alzheimer’ disease, mild cognitive impairment, subjective cognitive decline, temperament, clinical trials, recruitment

## Abstract

The prodromal stages of Alzheimer’s disease (AD) are the primary focus of research aimed at slowing disease progression. This study explores the influence of affective temperament on the motivation of people with mild cognitive impairment (MCI) and subjective cognitive decline (SCD) to participate in clinical trials. One hundred four subjects with MCI and SCD were screened for participation in pharmacological and non-pharmacological trials. Affective temperament was assessed based on the Temperament Evaluation of the Memphis, Pisa, Paris and San Diego (TEMPS) scale. Demographic variables and temperament subscales scores were compared between MCI and SCD patients and among patients participating in the pharmacological trial, the non-pharmacological trial and refusing participation. Twenty-one subjects consented to participate in the pharmacological trial, seventy consented to the non-pharmacological trial and thirteen refused to participate in any trial. Patients with SCD had greater education and more depressive temperamental traits than those with MCI. While older age, higher education and anxious temperament were negatively associated with participation in the pharmacological trial, irritable temperamental positively predicted pharmacological trial participation. In conclusion, temperamental features may affect the willingness of patients with MCI and SCD to take part in clinical trials and, especially, the choice to participate in pharmacological studies.

## 1. Introduction

The prevention and slowdown of cognitive impairment have assumed increasing importance. There is a critical need to develop pharmacological and non-pharmacological treatments that could be effective in the prodromal phases of Alzheimer’s disease (AD), in particular mild cognitive impairment (MCI) and subjective cognitive decline (SCD), to delay the onset of dementia [1]. MCI, by definition, is a condition characterized by impaired performance in one or more cognitive domains [2,3,4]. Although patients with MCI can maintain their independence in functional abilities, they are typically concerned about cognitive worsening [5,6,7]. In the literature, there is consensus that MCI is a clinical profile with a high probability of developing AD or another dementia [8,9,10,11,12,13,14]. On their count, patients with SCD are defined as subjects who complain of memory issues but do not exhibit any impairment at neuropsychological assessment [15]. Although there is heterogeneity among the studies, many authors have suggested that SCD may be associated with an increased risk of underlying AD [16,17,18,19,20,21,22]. A longitudinal study highlighted that SCD subjects concerned about their memory decline were at higher risk of developing dementia compared to those who reported SCD without concern [23].

Given the importance of recruiting people with prodromal AD symptoms, future clinical trials must consider the factors that motivate patients with MCI and SCD to participate in research. The availability of transportation services offered by medical facilities, home visits, remuneration and a favorable attitude toward medical research have been found to be strongly associated with interest in enrolment in a sample of patients with and without cognitive impairment [24]. Another study showed that older adults without dementia were motivated to take part in trials by the opportunity to learn more about their condition and the possibility of improving their own and other people’s health [25]. Moreover, it was found that the attitude of clinical trial participants increased across the dementia continuum, from normal cognition to AD. Older adults with cognitively normal function and MCI participants were most motivated by altruism, whereas AD patients were primarily driven by ambition to discover a therapy that would help their condition [26].

When personality traits were investigated in the context of routine health screening, conscientiousness was associated with greater adherence to the program and a higher frequency of follow-up visits for adult subjects [27], whereas high openness was a positive predictor of participation for elderly patients [28]. Instead, studies on the influence of psychological variables, such as anxiety traits, doubtfulness and fear of illness, on cancer screening behavior have not produced consistently reliable results. It is not clear whether the presence of these features facilitates or obstructs cancer screening and adherence to monitoring [29,30,31,32].

Although there are many studies on variables that affect involvement in screening and prevention, affective temperaments as motivating factors have not yet been investigated. Broadly speaking, temperament refers to individual constitutional differences in emotional and behavioral response that manifest early in life and remain relatively stable over time [33]. According to Hagop S. Akiskal, temperament determines emotionality, reactivity, impulse control and changes in energy levels and circadian rhythms [34]. Five effective temperaments were recognized in Akiskal’s model, namely depressive, hyperthymic, cyclothymic, irritable and anxious temperaments. Hyperthymic temperament is characterized by high energy levels, high emotional intensity, little need for sleep, extroversion, good self-esteem and resilience. The depressive temperament is related to stably low mood, low energy levels, hypersomnia, introversion, low self-esteem, brooding, indecision and self-disciplining [35,36]. Rapid changes in mood, energy levels, need for sleep, self-esteem and attitudes towards other people are the main features of the cyclothymic temperament. These fluctuations may be spontaneous or caused by life events subjectively perceived as stressful [36]. Irritable temperament is characterized by high energy levels and a tendency to be litigious and aggressive. In contrast to hyperthymic subjects, those with irritable temperaments tend to be more complaining and brooding. Finally, the anxious temperament is related to a high level of sympathetic activity, fear of disease, hypersensitivity to separation, difficulty in leaving a familiar environment, need for reassurance and hypersensitivity to medications [35,37,38,39]. Extensive literature has explored the influence of affective temperaments on clinical manifestations and the course of psychiatric disorders [37,40,41], but a relationship between the temperamental subtype and professional career choice has also been reported [42,43,44].

Since temperamental features appear to be important in everyday choices, we hypothesized they might have a role in trial decision-making. In this study, we focused on examining the temperamental traits of individuals experiencing cognitive difficulties who demand medical help. Further, we investigated whether individuals with MCI and SCD were more likely to adhere to clinical trials depending on their temperamental features and if affective temperament influenced their decision to participate in pharmacological trials rather than non-pharmacological trials.

## 2. Materials and Methods

### 2.1. Subjects 

Our sample consisted of 104 subjects (60 diagnosed with MCI and 44 with SCD) referred to the Memory Unit of the Neurological Clinic at Pisa University Hospital between January 2019 and December 2019 and screened for participation in a pharmacological and a non-pharmacological clinical trial. The pharmacological trial aimed to test monoclonal antibody treatment in people at a high risk of developing Alzheimer’s disease, including both MCI and SCD patients. The non-pharmacological study assessed the effectiveness of combined cognitive and physical training in slowing the progression of dementia. Exclusion criteria of both studies comprised any evidence of neurological pathologies, epilepsy, advanced neoplasia, recent cranial trauma, drug addiction, clinical evidence of depression or other psychiatric disorders according to the Diagnostic and Statistical Manual of Mental Disorders, 5th edition (DSM-5) [45]. Exclusionary diagnoses were based on clinical records and neuropsychiatric examination. All patients aged between 65 and 80 years with at least five years of education and with MCI or SCD confirmed at the neurological examination according to the current guidelines were proposed in both trials and were free to decide which one to participate in. Subjects did not obtain any economic or personal benefits from research participation. All participants signed free and informed consent previously approved by the institution’s ethics committee, and all data included in this manuscript were obtained in compliance with the Helsinki Declaration.

### 2.2. Procedure

All subjects underwent a complete neuropsychological evaluation to assess several cognitive domains and a clinical evaluation, including the collection of medical history and a complete neurological examination. The diagnosis of MCI was obtained according to current diagnostic criteria [6], and SCD subjects were classified using current guidelines [15]. Notably, the diagnosis of SCD required the presence of a self-experienced persistent decline in cognitive capacity compared with a previously normal status and unimpaired performance on standardized cognitive tests used to classify MCI or prodromal AD [15].

Demographic data, including age, gender and education years, were collected for all patients. Two self-report short Italian versions of the Temperament Evaluation in Memphis, Pisa and San Diego (TEMPS) scale were used to assess affective temperamental traits. The short TEMPS-Autoquestionnaire (TEMPS-A) [46], comprising 39 items with “yes” or “no” answers, was administered to 54 subjects, of which 35 were diagnosed with MCI and 19 with SCD. The brief TEMPS-Münster (TEMPS-M) [47], comprising 35 items with 1 to 5 Likert-type responses, was filled in by the latter 50 patients, of which 25 were diagnosed with MCI and 25 with SCD. Both questionnaires included five subscales, one for each affective temperamental disposition according to Akiskal’s model, measuring depressive, cyclothymic, hyperthymic, irritable and anxious temperamental traits. To aggregate data from both subsamples, given the lack of standardized norms for the general population allowing the conversion of raw scores into standardized ones, percentage scores were computed for each subscale, dividing raw scores by the maximum score achievable (e.g., a raw score of 28 in the anxious temperament subscale from TEMPS-M was converted into a percentage score of 80%, that is 28/35, given that the subscale was composed of 7 items with a maximum score of 5).

### 2.3. Statistical Analysis

Descriptive statistics were used to describe the demographic characteristics of the whole sample (*n* = 104). First, demographic variables and temperament subscales scores were compared between patients diagnosed with MCI and those diagnosed with SCD. Patients participating in pharmacological and non-pharmacological trials or refusing participation were then compared in the whole sample and the MCI group. Given the small number of patients with SCD refusing participation in trials (*n* = 2), only pharmacological and non-pharmacological trial groups were compared among patients with SCD. Pearson’s chi-squared test or, when appropriate, Fisher’s exact test were used for the comparison of categorical variables, with pairwise Fisher’s exact test used for post hoc comparisons. The Wilcoxon rank sum test and Kruskal–Wallis test were used, respectively, for two- and three-group comparisons of continuous variables, after excluding normality using Shapiro–Wilk test. The Dunn test was used for post hoc analyses. Finally, all variables, including diagnosis of SCD vs. MCI, were entered into a logistic regression model predicting participation in the pharmacological trial vs. adhesion to the non-pharmacological trial/refusal to participate. Backward stepwise model selection, based on minimizing the Akaike information criterion (AIC), was then applied. All analyses were performed using R Statistical Software (Foundation for Statistical Computing, Vienna, Austria). A statistical significance level of *p* < 0.05 was set in the analyses. False discovery rate (FDR) correction was applied to post hoc tests.

## 3. Results

The sample comprised 104 patients, of which 60 were diagnosed with MCI and 44 with SCD. The mean age of the whole sample was 73.94 ± 4.81 years (median = 74, interquartile range [IQR] = 71–77, range = 64–84), and 58 patients (55.77%) were females. Education years were, on average, 12.48 ± 4.29 (median = 13, interquartile range [IQR] = 8–17, range = 5–19). Compared to patients with MCI, those with SCD had significantly more education years and showed a significantly higher depressive temperament score (Table 1).

Overall, 21 subjects (10 with MCI and 11 with SCD) consented to participate in the pharmacological study, and 70 individuals (39 with MCI and 31 with SCD) adhered to the non-pharmacological trial. Thirteen patients (11 with MCI and 2 with SCD) refused participation in the proposed clinical trials. The three groups differed significantly in anxious temperament (Table 2), with patients participating in the pharmacological trial showing a significantly lower score than those participating in the non-pharmacological trial (p_FDR_ = 0.001) and those refusing participation (p_FDR_ = 0.004).

The same finding was confirmed among patients diagnosed with MCI (Table 3). Even in this case, patients participating in the pharmacological trial showed a significantly lower anxious temperament score with respect to those participating in the non-pharmacological trial (p_FDR_ = 0.019) and those refusing participation (p_FDR_ = 0.014).

In addition, among patients diagnosed with SCD, those participating in the pharmacological trial were more frequently male, had a significantly higher score on the irritable temperament subscale and showed a significantly lower anxious temperament score compared to patients participating in the non-pharmacological trial (Table 4).

Finally, according to the multivariate logistic regression model, including all variables, education years and anxious temperament score were negatively associated with participation in the pharmacological trial, while irritable temperamental traits showed an opposite effect (Table 5). After backward stepwise selection, age, anxious and irritable temperament scores were retained as significant predictors, with the former two showing a negative association with participation in the pharmacological trial and the latter a positive effect (Table 5).

## 4. Discussion

In this study, we explored if there were any differences in the affective temperaments of MCI and SCD patients involved in pharmacological and non-pharmacological clinical trials. Our results showed no differences in age and gender between SCD patients and MCI patients who were referred to the Memory Unit of the Neurological Clinic. In line with a previous study in which SCD patients had a higher education than MCI patients [48], our SCD patients reported more years of school attendance than those with MCI. While other temperamental traits did not differ between the groups, more depressive traits were observed in patients with SCD compared to MCI.

Depressive temperamental traits may likely have a role in how patients perceive their memory abilities. Subjective memory complaints, in addition to being recognized as a predictor of cognitive decline and AD, have already been associated with depression and specific personality traits [49]. The relationship between depressive symptoms and memory complaints has been reported in several studies [50,51,52,53], and distractibility, lack of concentration and poor memory are considered core depressive symptoms [45]. Consequently, memory complaints in SCD subjects could result from a tendency to negatively evaluate their own memory due to depressive personality features. Personality measures such as low feelings of mastery and perceived self-efficacy, higher neuroticism [49,54], low levels of self-esteem [54], hypochondriasis and psychasthenia symptoms have already been associated with subjective memory complaints in older adults without memory deficits [55].

Anxious temperament appears to discourage participation in pharmacological studies but not in non-pharmacological ones. In our sample, patients with MCI and SCD participating in non-pharmacological trials showed significantly more anxious traits than those participating in pharmacological trials. Our results are consistent with previous reports for cancer screening, observing that anxious traits may not necessarily favor participation in studies [29,31,32]. Moreover, non-pharmacological studies, possibly thought to be less risky, seem to be preferred by patients with anxious traits.

Conversely, irritable temperament tends to be associated with participation in pharmacological trials, at least in patients with SCD. To the best of our knowledge, no previous study investigated the relationship between irritable temperament or, more generally speaking, irritability and participation in clinical research. Irritable temperament has been previously associated with trait impulsivity [56]. Moreover, in adolescents, irritability has been linked to temporal discounting impulsivity, the propensity to choose smaller immediate rather than larger delayed rewards [57]. Accordingly, we hypothesized that people with more pronounced irritable traits might prefer rapidly acting through riskier treatment options (e.g., pharmacological treatments) against slower and more demanding interventions (e.g., psychological treatments).

Notably, anxious and irritable temperaments had divergent effects. Both temperamental features were found to mainly affect participation in the pharmacological trial. Indeed, anxious temperament prevented participation in the pharmacological trial but did not distinguish patients refusing participation in any trial from those taking part in the non-pharmacological one. On the other hand, irritable temperament was associated with participation in pharmacological research and, among SCD patients, distinguished between subjects adhering to the pharmacological vs. the non-pharmacological trial. Although modest positive associations were observed between anxious and irritable temperament in the general population (r = 0.24–0.28) [46,47], the discrepancy between their influences on trial participation may be explained by different mediating variables to be further explored, such as harm avoidance for anxious temperament and impulsivity for the irritable one.

Older age, high education and anxious temperament are negative predictors of participating in a pharmacological trial, whereas having an irritable temperament is a positive predictor of participation in the pharmacological trial versus the non-pharmacological trial. Age was found to be a poor predictor of willingness to participate in a medical treatment trial in prior research on elderly individuals, with the intention to participate declining with age [58]. Older adults, members of racial and ethnic minorities and those with low-income levels generally tended to have reduced motivation to participate in pharmacological trials, according to prior studies [59,60,61]. Conversely, the role of educational level is not unequivocally established. In our study, education years were negatively associated with participation in the pharmacological trial only in the logistic regression model, including all variables, while no significant differences were detected in the comparative analyses nor was educational level selected among predictors of pharmacological trial participation after stepwise regression. Similarly, mixed findings were found in previous studies. Individuals who agreed to take part in a mock clinical study of a drug differed from those who declined based on their gender but not on their educational level [62]. High educational levels may also boost willingness to participate in pharmacological and non-pharmacological research according to certain studies [63,64], but other individual and environmental factors, including race and attitude toward research (e.g., trust level and perceived societal benefits), may have a stronger influence on the prediction of study participation [65]. We hypothesized that the effect of educational level might interact with other variables, including the type of disease, temperament and personality characteristics, as well as trial features. On the one hand, higher education may be associated with a greater trust in research, while on the other, highly educated patients may have a greater awareness of pharmacological risks. Further studies could help unravel potentially meaningful moderators and mediators of education effects on trial participation.

Our results are consistent with research investigating psychological factors that affect patients’ willingness to participate in phase I, II and III pharmacological trials. High exploratory tendency and perceived self-efficacy were observed in volunteers for phase I studies [66]. Moreover, fewer anxiety traits and social avoidance were previously found in patients willing to participate in phase II and III clinical trials compared to those who refused [67]. As it is well known, anxious dispositions may result in the avoidance of perceived threats, including social contexts as well as unfamiliar situations, drug intake and medical procedures. Social avoidance may thus be indicative of a general anxious and avoidant attitude that can influence pharmacological research participation.

Identifying an individual’s temperamental and personality features that affect their decision to participate in a clinical trial needs to be considered during the creation of recruitment and communication strategies. Particularly, the involvement of psychologists and social sciences experts can help overcome self-selection bias using personalized communication. Attempting to enroll the greatest number of patients, including those with personality traits that would prevent them from participating, would increase the overall number of participants and result in a more representative sample.

Several methodological limitations of our study should be considered. A first limitation comes from the clinical setting in which patients were recruited, a tertiary referral neurological unit, where more complicated patients could have been overrepresented, thus undermining the representativeness of the sample and, together with the limited sample size, the generalizability of the results. Second, temperament was assessed using self-reported questionnaires that may be subjected to response biases due, for instance, to perceived social desirability, lack of introspective ability, influence of current affective states and exaggeration of symptoms to obtain assistance. Moreover, two different versions of the TEMPS scale were used, and scores were subsequently aggregated, limiting the comparability of our results with other studies. Further investigations are required to confirm our findings using informant-reported or clinician-rated instruments, possibly with established standardized norms, to assess affective temperament. Finally, although psychiatric disorders were considered among the exclusion criteria, no systematic, structured evaluation of psychopathology was used, making it possible that some patients with undiagnosed psychiatric conditions, such as patients with anxiety disorders scoring higher on anxious temperamental traits, were included. Whether psychopathology may interact with temperament variables to influence trial participation still needs to be explored.

## 5. Conclusions

According to our preliminary findings, depressive temperament may be the reason for an early search for medical assistance in SCD patients since it increases the perception of how invalidating cognitive symptoms are. Temperamental features may also influence SCD and MCI patients’ willingness to participate in clinical trials. Participation in pharmacological studies may be encouraged by irritable temperament but discouraged by anxious temperamental traits. Temperament should be considered while designing clinical trials for individuals with prodromal AD because it influences health behavior and decision-making and plays a significant role in the attitude of patients towards participation. Building multidisciplinary teams, including clinicians, psychologists and social sciences experts, could help tailor communication strategies to each patient during the recruitment phase. For instance, a different communication approach may be required for patients showing highly anxious traits compared to those with predominantly irritable temperamental features. Further studies are warranted to develop temperament-sensitive recruitment procedures to include samples representative of the entire target population.

## Figures and Tables

**Table 1:** Differences between mild cognitive impairment (MCI) and subjective cognitive decline (SCD) patients.

	MCI (*n* = 60)	SCD (*n* = 44)		
	Mean ± SD/*n* (%)	Mean ± SD/*n* (%)	r	*p*
Age (years)	74.18 ± 4.5	73.61 ± 5.2	0.08	0.446
Sex (male)	29 (48.3%)	17 (38.6%)	0.61	0.433
Education (years)	11.45 ± 4.2	13.89 ± 4.1	−0.30	0.002
Depressive temperament	24.43 ± 21.1	34.9 ± 21.8	−0.23	0.018
Cyclothymic temperament	26.47 ± 20.5	26.46 ± 19.9	−0.01	0.953
Hyperthymic temperament	44.7 ± 19.2	42.53 ± 18.9	0.04	0.711
Irritable temperament	9.91 ± 12.9	14.33 ± 13.6	−0.18	0.073
Anxious temperament	35.14 ± 30.4	33.39 ± 27.3	0.03	0.733

MCI—mild cognitive impairment; SCD—subjective cognitive decline; SD—standard deviation.

**Table 2:** Differences between patients participating in pharmacological (PT) and non-pharmacological trials (NPT) or refusing participation (RP).

	PT (*n* = 21)	NPT (*n* = 70)	RP (*n* = 13)			
	Mean ± SD/*n* (%)	Mean ± SD/*n* (%)	Mean ± SD/*n* (%)	χ^2^	*p*	Post Hoc
Age (years)	72.38 ± 4.5	74.53 ± 4.8	73.31 ± 5.2	3.25	0.197	-
Sex (male)	13 (61.9%)	29 (41.4%)	4 (30.8%)	3.84	0.147	-
Education (years)	11.81 ± 5.0	12.83 ± 4.0	11.69 ± 4.8	0.94	0.624	-
Diagnosis (MCI)	10 (16.7%)	39 (65.0%)	11 (18.3%)	4.85	0.089	-
Depressive temperament	28.57 ± 20.1	29.29 ± 22.0	27.06 ± 25.4	0.29	0.863	-
Cyclothymic temperament	20.92 ± 17.1	28.04 ± 21.3	26.92 ± 17.6	1.73	0.420	-
Hyperthymic temperament	47.28 ± 13.3	41.96 ± 19.2	47.94 ± 25.2	1.67	0.434	-
Irritable temperament	17.18 ± 14.4	11.02 ± 13.1	7.14 ± 10.7	5.81	0.055	-
Anxious temperament	14.46 ± 8.2	38.88 ± 29.9	42.49 ± 32.7	14.65	0.001	PT < NPT, RP

PT—pharmacological trial; NPT—non-pharmacological trials; RP—refusing participation; MCI—mild cognitive impairment; SD—standard deviation.

**Table 3:** Differences between mild cognitive impairment (MCI) patients participating in pharmacological (PT) and non-pharmacological trials (NPT) or refusing participation (RP).

	PT (*n* = 10)	NPT (*n* = 39)	RP (*n* = 11)			
	Mean ± SD	Mean ± SD	Mean ± SD	χ^2^	*p*	Post Hoc
Age (years)	73.2 ± 5.4	74.51 ± 4.1	73.91 ± 5.3	0.32	0.853	-
Sex (male)	5 (50.0%)	20 (51.3%)	4 (57.1%)	-	0.697	
Education (years)	10.3 ± 4.7	11.59 ± 3.9	12 ± 4.7	0.80	0.671	-
Depressive temperament	20 ± 16.9	26.01 ± 21.0	22.89 ± 25.5	0.78	0.676	-
Cyclothymic temperament	18.21 ± 16.0	29.43 ± 22.2	23.48 ± 16.0	1.85	0.396	-
Hyperthymic temperament	42.86 ± 11.7	44.37 ± 18.6	47.56 ± 27.0	0.26	0.880	-
Irritable temperament	11.43 ± 12.1	10.58 ± 13.8	6.17 ± 10.0	1.53	0.464	-
Anxious temperament	11.07 ± 7.2	38.77 ± 30.0	44.16 ± 35.5	8.66	0.013	PT < NPT, RP

PT—pharmacological trial; NPT—non-pharmacological trials; RP—refusing participation; SD—standard deviation; MCI—mild cognitive impairment.

**Table 4:** Differences between subjective cognitive decline (SCD) patients participating in pharmacological (PT) and non-pharmacological trials (NPT).

	PT (*n* = 11)	NPT (*n* = 31)		
	Mean ± SD	Mean ± SD	r	*p*
Age (years)	71.64 ± 3.6	74.55 ± 5.6	−0.25	0.105
Sex (male)	8 (72.7%)	9 (29.0%)	-	0.029
Education (years)	13.18 ± 5.1	14.39 ± 3.5	−0.05	0.760
Depressive temperament	36.36 ± 20.3	33.41 ± 22.9	0.04	0.796
Cyclothymic temperament	23.38 ± 18.5	26.31 ± 20.4	−0.05	0.752
Hyperthymic temperament	51.3 ± 14.0	38.94 ± 19.8	0.29	0.063
Irritable temperament	22.4 ± 14.7	11.58 ± 12.3	0.33	0.036
Anxious temperament	17.53 ± 8.1	39.02 ± 30.4	−0.34	0.029

SCD—subjective cognitive decline; PT—pharmacological trial; NPT—non-pharmacological trials; SD—standard deviation.

**Table 5:** Multivariate logistic regression models predicting participation in the pharmacological trial vs. non-pharmacological trial/refusal.

	A. Full Multivariate Model (AIC = 89.76, R^2^_adj_ = 0.26)			B. Backward Stepwise Selected Model (AIC = 84.80, R^2^_adj_ = 0.22)		
Variables	Estimate	OR (95% CI)	*p*	Estimate	OR (95% CI)	*p*
(Intercept)	11.47	-	0.068	10.21	-	0.037
Age (years)	−0.14	0.87 (0.75–1.01)	0.068	−0.15	0.87 (0.76–0.98)	0.027
Sex (male)	0.72	2.06 (0.59–7.21)	0.258	-	-	-
Education (years)	−0.16	0.85 (0.73–0.99)	0.041	-	-	-
Diagnosis (MCI)	−0.76	0.47 (0.13–1.73)	0.256	-	-	-
Depressive temperament	0.02	1.02 (0.98–1.06)	0.403	-	-	-
Cyclothymic temperament	−0.03	0.97 (0.93–1.02)	0.230	-	-	-
Hyperthymic temperament	−0.00	0.10 (0.96–1.04)	0.867	-	-	-
Irritable temperament	0.05	1.05 (1–1.10)	0.048	0.05	1.05 (1.01–1.09)	0.026
Anxious temperament	−0.08	0.93 (0.88–0.98)	0.003	−0.07	0.93 (0.90–0.97)	0.001

AIC—Akaike information criterion; OR—odds ratio; MCI—mild cognitive impairment.

## Data Availability

The data presented in this study are available on request from the corresponding author. The data are not publicly available due to privacy or ethical restrictions.
